# Evaluation of Surgical Strategy Based on the Intraoperative Superior Oblique Tendon Traction Test

**DOI:** 10.1371/journal.pone.0168245

**Published:** 2016-12-16

**Authors:** Miwa Komori, Hiroko Suzuki, Akiko Hikoya, Mayu Sawada, Yoshihiro Hotta, Miho Sato

**Affiliations:** Department of Ophthalmology, Hamamatsu University School of Medicine, Hamamatsu, Japan; Universita degli Studi di Firenze, ITALY

## Abstract

**Purpose:**

To clarify the efficacy of a surgical strategy based on the superior oblique tendon traction test.

**Methods:**

A retrospective chart review was performed between January 2002 and June 2015. During that period, a single inferior oblique muscle (IO) myectomy and a combined IO myectomy and superior oblique muscle (SO) tuck procedure were performed based on SO tendon looseness as revealed by a traction test. The surgical effects of both procedures and the number of operations were analyzed.

**Results:**

Sixty-five cases were retrieved. Seventy-four surgeries were required. The IO myectomy and simultaneous groups included 48 and 17 cases, respectively. Pre-operative vertical deviation was significantly lower in the IO myectomy (11.8 prism diopters) than in the simultaneous (27.2 prism diopters; Mann–Whitney *U*-test, *P* < 0.001) group. The mean induced changes were 9.4 prism diopters and 21.6 prism diopters in the IO myectomy and simultaneous groups, respectively, and the postoperative vertical deviation was not significantly different. On average, 1.13 and 1.18 surgeries per patient were performed in the IO myectomy and simultaneous groups, respectively.

**Conclusion:**

The simultaneous surgery of inferior oblique myectomy and superior oblique tuck is safe and effective for treating large angle of congenital/idiopathic superior oblique palsy with a lax superior oblique tendon, as determined by the traction test.

## Introduction

Congenital superior oblique palsy (SOP) is the most common cause of vertical deviation in children. These patients often show large vertical deviation and abnormal head posture. The inferior oblique muscle (IO) weakening procedure is often performed as a first-line therapy [1], but it is not always successful. Additional procedures, such as superior oblique muscle (SO) tuck, superior rectus muscle (SR) recession, contralateral inferior rectus muscle (IR) recession, or IO anterior transposition, have been proposed. The main complications of an SO tuck are iatrogenic Brown syndrome [2, 3] and late undercorrection [4, 5]. The complications of rectus muscle recession include both late overcorrection [6, 7] and late undercorrection [8], as well as eyelid retraction [9].

**Fig 1:**
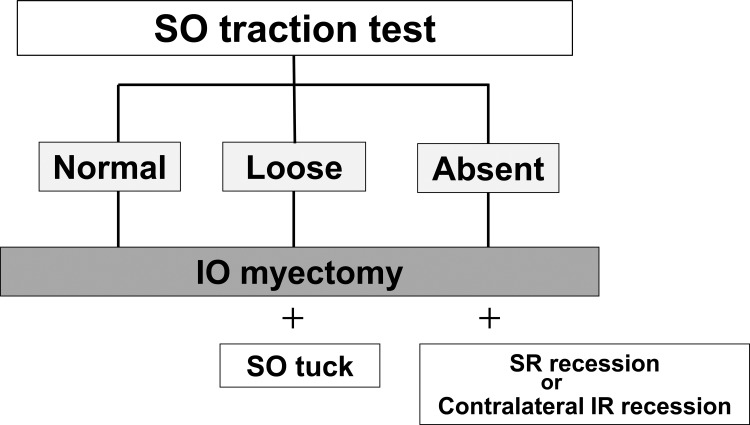
Procedures for congenital superior oblique muscle palsy. SO: superior oblique muscle; IO: inferior oblique muscle; SR: superior rectus muscle; IR: inferior rectus muscle

We determined the procedures to be performed by following the algorithm proposed by Plager (Fig 1) [10]. The algorithm recommends IO myectomy be performed alone in cases with normal tendon laxity of the SO. In cases with a lax SO tendon, a procedure involving simultaneous IO myectomy and SO tuck is performed until tendon laxity matches that on the contralateral side in a traction test. In cases with a lax tendon, the vertical deviation often exceeds 15 prism diopters (PD), and they are unlikely to be corrected via the IO myectomy procedure alone. Previous literature [2, 11, 12] reported that multiple SOP surgeries were required on patients with lax SO tendon. However, there are few detailed studies [4, 13] of a simultaneous IO myectomy and SO tuck procedure for congenital SOP.

The purpose of this study is to clarify the efficacy of a surgical strategy based on superior oblique tendon looseness, which recommends an SO tuck procedure along with IO myectomy for lax SO tendon patients.

## Methods

A retrospective chart review was performed to retrieve information concerning patients who underwent IO myectomy with or without SO tuck between January 2002 and June 2015 at the Hamamatsu University School of Medicine under the diagnosis of congenital or idiopathic SOP. The definition of congenital or idiopathic SOP is as follows: 1) early or unclear onset of vertical deviation with abnormal head posture associated with a positive Bielschowsky head tilt test; 2) over-elevation in adduction on the paretic eye; 3) unilateral small SO muscle on coronal magnetic resonance imaging (MRI) when available, or cases that are difficult to diagnose; and 4) mainly vertical, not torsional, diplopia if it is present at all. The following patients were excluded from the study: those who were too young to assess the angle of preoperative deviation accurately, those for whom no one-month follow-up was conducted, those who had previous surgical histories, bilateral SOP, or those who had undergone ipsilateral SR recess or contralateral IR recess. In order to ensure the effectiveness and safety of the simultaneous surgery, the surgical results were evaluated in two groups: an IO myectomy alone group and a simultaneous group. The simultaneous procedure included both an IO myectomy and an SO tuck procedure. We included some patients who underwent horizontal muscle surgeries concurrently with the IO myectomy or simultaneous surgery. When the tendon was not found, we labeled this as SO tendon absent, and these cases were not included in this study. Post-operative follow-up was conducted for patients for at least 1 month.

Pre- and post-operative vertical deviation were measured with a prism and cover test at a distance, and stereoacuity was measured with a Stereo Fly Test (Stereo Optical Co. Inc., Chicago, IL, USA). Pre- and post-operative over-elevations in adduction were recorded as an IO function classified from +4 to –4. Numeric ratings were used to describe the degree of oblique dysfunction: +1 to +4 indicating minimal to maximal overaction, and -1 to -4 indicating minimal to maximal underaction [14]. If there was no IO dysfunction, the score was 0.

Pre- and post-operative abnormal head posture was collected from both the clinical chart and from the pre- and post-operative photographs taken under natural viewing conditions.

All the surgeries were performed under general anesthesia with surgical microscopes. The IO myectomy was performed by multiple surgeons in one institution under the supervision of the senior ophthalmologists by M.S. and A.H. The combined surgeries were performed by M.S. and A.H.

The traction test of the SO tendon was performed on all cases under general anesthesia before the procedures were decided. The traction test was performed as follows [15, 16]: Briefly, the eye was pulled in and up and was rotated over the SO tendon. When the tendon felt very loose (-3 to -4 looseness), we performed the SO tendon tuck, along with the IO myectomy. When the SO tendon felt normal or mildly loose (-1 to -2 looseness), or there was no difference between the eyes, we performed IO myectomy alone.

The ipsilateral IO was weakened by myectomy in all patients. The IO myectomy procedure was performed with a fornix incision. The IO was hooked completely at its insertion site, and approximately 5 mm of the muscle was resected. Its cut end was confirmed to be pulled into Tenon’s capsule. The SO tendon tuck began with the superior-temporal fornix conjunctiva incision. After the SR was identified, the sclera of the temporal border of the SR muscle was carefully searched to identify the SO tendon. When the tendon was not found in that area, then the nasal border of the SR was searched. When the tendon was found, it was tucked with a double-armed 5–0 polyester suture, and the tip of the tucked tendon was sutured onto the sclera. When the tendon was found on the abnormal location, it was sutured to the normal location. After tucking the SO tendon, the traction test and the fundus examination were performed to determine the amount of the tuck.

The overall success was determined with respect to the need for additional operations during the follow-up period. We classified patients who underwent one session as the success group, and the patients who required additional sessions were categorized as the failure group. When the patient showed minor vertical deviation or residual abnormal head posture, but either the patient or the guardian did not wish to have the second procedure, the procedure was classified as a success.

A Mann-Whitney *U*-test was used for comparisons between the IO myectomy and simultaneous groups. A *P*-value less than 0.05 was considered statistically significant.

This study protocol was reviewed and approved by the institutional review board of the Hamamatsu University School of Medicine (No. E16-021). The institutional review board waived the need for a written consent from the participants. Patient information was anonymized and de-identified prior to analysis. All study conduct adhered to the tenets of the Declaration of Helsinki.

## Results

During this period, 185 patients were diagnosed as having SOP. Among these, 62 were diagnosed as having acquired SOP and 123 were diagnosed as having congenital or idiopathic SOP. The following patients were excluded from the study: those who were too young to accurately assess the angle of preoperative deviation (10 cases), those for whom there was no follow-up after 1 month (2 cases), those with a previous surgical history (16 cases) or bilateral SOP (6 cases), and those who underwent ipsilateral SR recess or contralateral IR recess (24 cases), including three patients in whom the SO tendon was absent. Finally, 65 cases were retrieved. The IO myectomy and simultaneous groups included 48 and 17 cases, respectively. Horizontal muscle surgeries were performed for 4 cases in the IO myectomy group and one case in the simultaneous group concurrently with the IO myectomy or simultaneous surgery. Patient ages ranged between 2 and 71 years (mean: 10.3 years) in the IO myectomy group and 1 and 45 years (mean: 11.2 years) in the simultaneous group. The follow-up period was between 1 and 142 months post-operatively (mean: 18.3 months) in the IO myectomy group and between 3 and 89 months post-operatively (mean: 19.2 months) in the simultaneous group. There was no statistically significant difference between the two groups in terms of either age (p = 0.221) or follow-up period (p = 0.288, Mann-Whitney *U*- test). The SO tendon tuck size ranged from 4 to 14 mm. In 2 cases, the SO tendons were transposed to their normal locations. There were no cases of amblyopia.

### Vertical deviations

**Fig 2:**
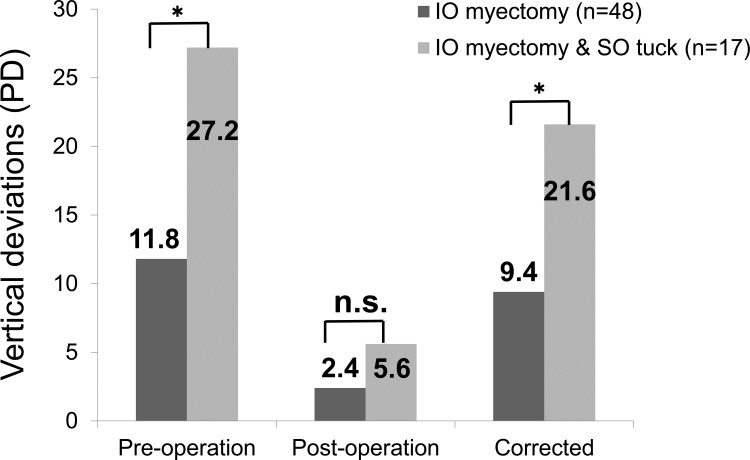
Vertical deviations of pre-and post-operation and corrected values. PD: prism diopters; SO: superior oblique muscle; IO: inferior oblique muscle, * *p* < 0.01 (Mann-Whitney U-test), n.s.: not significant

Pre-operative vertical deviation was significantly lower in the IO myectomy (11.8 PD [standard deviation (SD): 6.0 PD]; range: 0–25 PD) than in the simultaneous (27.2 PD [SD: 7.6 PD]; range: 8–40 PD; Mann-Whitney *U*-test, *P* < 0.001) group. However, these values were not significantly different post-operatively (2.4 PD [SD: 5.8 PD]; range: -18-16 PD, and 5.6 PD [SD: 11.1 PD]; range: -18-35 PD, respectively; Mann-Whitney *U*-test, *P* = 0.069). The mean induced change in vertical deviation was 9.4 PD (SD: 7.2 PD); range: -4-28 PD and 21.6 PD (SD: 13.9 PD); range: -5-48 PD in the IO myectomy and simultaneous groups, respectively, and these values were significantly different (Mann-Whitney *U*-test, *P* < 0.001) (Fig 2).

**Fig 3:**
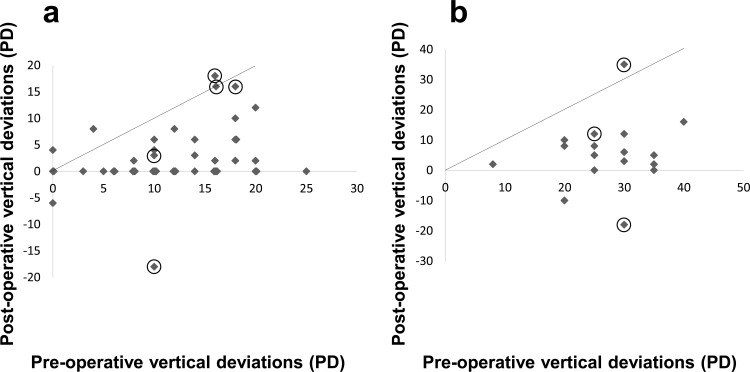
Pre- and post-operative vertical deviations in each patient who underwent. **a)** an inferior oblique muscle (IO) myectomy alone (n = 48); and **b)** a simultaneous IO myectomy and superior oblique muscle tuck procedure (n = 17). Circles indicate patients who required re-operations. PD: prism diopters

Fig 3 shows pre- and post-operative vertical deviations for each patient who underwent IO myectomy alone and the simultaneous procedure. Most cases, both in the myectomy and simultaneous groups, came within 10 PD vertical deviations post-operatively.

### Stereoacuity

Stereoacuity was measured with a Stereo Fly Test both pre- and post-operatively for 38 cases in the IO myectomy group and 17 cases in the simultaneous group. Ten patients in the IO myectomy group were too young for the stereoacuity assessment. In the IO myectomy group, 16 cases demonstrated improved stereoacuity, and 20 cases achieved better than 80 seconds of arc stereoacuity. No stereopsis remained in 6 cases post-operatively. In the simultaneous group, 10 of 17 cases had no stereopsis pre-operatively. Stereoacuity improved in 4 cases, and no stereopsis remained in 9 of 10 cases post-operatively (Table 1).

**Table 1:** Pre- and post-operative stereoacuity in the two groups.

IO myectomy (n = 38)					IO myectomy & SO tuck (n = 17)				
Pre-operation	Post-operation				Pre-operation	Post-operation			
	Fly(-)	3000"	800–100"	80–40"		Fly(-)	3000"	800–100"	80–40"
Fly(-)	6	2	2	1	Fly(-)	9	0	1	0
3000"	0	1	4	1	3000"	0	1	0	0
800–100"	0	0	2	6	800–100"	1	0	1	3
80–40"	0	0	1	12	80–40"	0	0	0	1

IO: inferior oblique muscle; SO: superior oblique muscle

### IO function

Pre- and post-operative IO functions in the two groups are shown in Table 2. All patients had pre-operative IO overaction. In the myectomy group, 7 cases showed persistent IO overaction; however, most cases showed an improvement, and only one case showed –2 of IO dysfunction. In the simultaneous group, 9 cases showed moderate dysfunction with the so-called Brown syndrome 1 week post-operatively; however, all except one patient showed an improvement 3 months after the surgery.

**Table 2:** Pre- and post-operative IO function in the two groups.

IO function		-4	-3	-2	-1	0	1	2	3	4
**Pre-operation**										
IO myectomy (n = 48)		0	0	0	0	0	12	25	11	0
IO myectomy & SO tuck (n = 17)		0	0	0	0	0	2	9	6	0
**Post-operation**										
IO myectomy (n = 48)	1 month	0	0	1	2	38	7	0	0	0
IO myectomy & SO tuck (n = 17)	1 week	0	0	9	4	4	0	0	0	0
IO myectomy & SO tuck (n = 17)	3 months	0	0	1	8	8	0	0	0	0

IO: inferior oblique muscle; SO: superior oblique muscle

### Head posture

The changes in abnormal head posture for the two groups are shown in Table 3. In the IO myectomy group, 30 patients achieved normal head posture restoration, and 13 experienced improved head posture; these were classified as the success group. Four patients had persistent abnormal head posture, and one had reversed head posture; these 5 patients underwent additional surgery and were considered to show poor outcomes. In the simultaneous group, 9 patients were cured, 4 patients showed an improvement, and there was one reversed patient; all of these were classified as being in the successful treatment group. Two cases with persistent abnormal head posture, and another case with reversed head posture, required additional surgeries. These 3 patients were considered to show poor outcomes.

**Table 3:** Changes in abnormal head posture for the two groups.

		IO myectomy (n = 48)		IO myectomy & SO tuck (n = 17)	
		n	(vertical deviations)	n	(vertical deviations)
**Success**					
	Cure	30	(1.5 PD)	9	(5.8 PD)
	Improve	13	(3.7 PD)	4	(6.3 PD)
	Reverse	0	(0 PD)	1	(-10.0 PD)
**Fail**					
	Persist	4	(13.3 PD)	2	(23.5 PD)
	Reverse	1	(-18.0 PD)	1	(-18.0 PD)

IO: inferior oblique muscle; SO: superior oblique muscle; PD: prism diopters

Post-operative mean vertical deviations are shown in brackets.

### Additional surgery

In the IO myectomy group, the secondary procedures for undercorrection were SO strengthening (3 cases) and IR recession of the contralateral eye, followed by the 3^rd^ surgery of IR advancement for one patient. The second surgery for overcorrection was IO myectomy of the contralateral eye. In the simultaneous group, one case with undercorrection underwent contralateral IR recession, and the other case underwent re-tucking of the SO and small recession of the ipsilateral SR. The case with overcorrection underwent release of the suture 1.5 years after the initial surgery.

### Success rate

In total, 74 surgeries were performed on 65 patients during the follow-up period. Fifty-four surgeries were performed on 48 patients in the IO myectomy group, and 20 surgeries were performed on 17 patients in the simultaneous group. The total success ratio was 89.6% (43 of 48 cases) in the myectomy group and 82.4% (14 of 17 cases) in the simultaneous group. On average, 1.13 and 1.18 surgeries per patient were performed in the IO myectomy and simultaneous groups, respectively, and 1.14 overall.

## Discussion

Several procedures are used to correct the large angle vertical deviation resulting from congenital/idiopathic SOP. Although several studies have examined simultaneous IO weakening and SO tendon tuck procedures, they deal either with acquired SOP [17, 18], or mixed origins [4]. In this study, we collected information on congenital/idiopathic SOP alone. Patients with congenital/idiopathic SOP have different characteristics from those with acquired SOP such as large fusional amplitude, no torsional diplopia, SO tendon laxity, SO muscle volume [19] or abnormal insertion site to the sclera [20, 21].

Congenital SOP patients often show tendon anomalies, including absent tendon; on the contrary, acquired SOP patients do not show tendon abnormalities [20]. It is also known that the loose SO tendon is usually accompanied by a small SO muscle belly [11]. Recently, Yang reported that congenital SOP patients often show absence of a trochlea nerve, and those without a trochlear nerve had a hypoplastic SO muscle belly [22, 23]. Therefore, the surgical approaches to SOP should be different between congenital/idiopathic and acquired SOP. The SO tendon tuck is especially useful for cases with lax tendons, because it does not disturb blood supply to the anterior part of the globe, and further surgery can be performed with fewer risks of anterior segment ischemia. If the SO tendon tuck is appropriately performed on the patient with a lax tendon, the risk of Brown syndrome can be reduced.

There is some controversy in the findings on the effects between IO myectomy and the graded IO recess procedure [24]. Bahl reported that IO myectomy is more effective than IO recession [25], Rajavi reported that there is no difference between IO myectomy and IO anterior transposition [26], and Ghazawy reported that IO myectomy has some advantage over IO anterior transposition [27]. The IO anterior transposition procedure is reported to be effective with a single muscle surgery to correct congenital or acquired SOP [28], but it also has serious complications in relation to anti-elevation syndrome [29] or eyelid shape deformity [30]. Lee reported the effectiveness of IO myectomy on unilateral SOP based on the MRI trochlear nerve findings, and concluded that patients without a trochlear nerve showed higher rates of undercorrection and recurrence of ipsilateral hypertropia after IO myectomy than those with a trochlear nerve [31].

The indications for 2 muscle surgeries for large angle SOP have not been established. Helveston reported single IO weakening is effective for 7.9 PD in the primary position [2], and Hatz reported 5 PD correction with IO recession and 11 PD anterior transposition in the primary position [32]. They recommend 2 muscle surgery in cases with greater than 15 PD in the primary position. Nejad reported up to 17.3 PD of correction with IO recession and recommended 2 muscle surgery in cases with greater than 20 PD in the primary position [33]. Caca reported that IO myectomy was effective for as much as 9.6 PD in the primary position and recommended 2 muscle surgeries in cases with greater than 25 PD in the primary position [34]. In our series, the mean induced change after IO myectomy alone was 9.4 PD in the primary position. The SO tuck procedure added 12.2 PD of correction in the primary position, and then only one patient showed a remaining vertical deviation over 20 PD. Additionally, using the intraoperative traction test to determine the amount of tuck is safe and useful in avoiding postoperative Brown syndrome.

Helveston reported an average of 1.26 surgeries were performed per patient on 190 SOP patients in his study [2]. Sato reported that 35 surgeries were required on 24 patients (1.46 surgeries per patient) who underwent IO weakening procedure as the first choice [11], and in the other series [12] she reported the surgical numbers based on tendon anomalies as follows: Class I, 1.27; Class II, 1.33; and Class III, 1.80. Compared to the previous reports, starting with a simultaneous surgery on loose tendon cases decreased the surgical numbers significantly (to an average of 1.13 and 1.18 surgeries per patient in the IO myectomy and simultaneous groups, respectively, and 1.14 overall).

The disadvantages of the SO tuck procedure are: 1) the inability to perform when the SO is absent, 2) the subjectivity of the traction test of the SO tendon and the need for more experience with it, and 3) the lateness of undercorrection. Despite these disadvantages, this procedure shows a crucial benefit in that we can reserve vertical rectus muscles for future surgeries.

This study has some limitations. As it is retrospective in nature, the definitions of “success” or “failure” are subjectively decided by parents and/or surgeons as a request for re-operation. If we use a clearer definition of success, the rate of failure may increase. However, it is also difficult to achieve perfect alignment in 9 gaze positions, or with the head tilted toward the opposite direction. We understand that the inferiority of the Stereo Fly Test is also a weakness of this study; however, within a clinical setting it is still useful for working with young children.

Nevertheless, our results indicate that the surgical strategy of using pre- and intraoperative SO tendon traction tests is useful for both selecting the patients who need 2 muscle surgeries and determining the necessary amount of SO tuck.
